# Developing and Evaluating A One-Stop Patient-Centered Interprofessional Collaboration Platform in Taiwan

**DOI:** 10.3390/healthcare8030241

**Published:** 2020-07-29

**Authors:** Hung-Jung Lin, Yen-Ling Ko, Chung-Feng Liu, Chia-Jung Chen, Jing-Jia Lin

**Affiliations:** 1 Department of Emergency Medicine, Chi Mei Medical Center, Tainan 71004, Taiwan; hjlin52@gmail.com; 2 Department of General Internal Medicine, Chi Mei Medical Center, Tainan 71004, Taiwan; dreamyfairy79@hotmail.com; 3 Medical Research Department, Chi Mei Medical Center, Tainan 71004, Taiwan; 4 Department of Information Systems, Chi Mei Medical Center, Tainan 71004, Taiwan; carolchen@mail.chimei.org.tw (C.-J.C.); shootme@mail.chimei.org.tw (J.-J.L.)

**Keywords:** healthcare, interprofessional collaboration, communication, patient-centered, computerized platform, resistance to change

## Abstract

(1) Background: Effective healthcare collaboration not only improves the outcomes of patients, but also provides benefits to healthcare providers. A patient-centered communication platform, a so-called “one-stop platform”, is necessary to promote interprofessional collaboration (IPC) for optimal patient care. (2) Methods: Chi Mei Medical Center developed a patient-centered computerized platform to fulfill interprofessional collaboration needs. The platform features a spiral-shaped integrated care area and a communication area that allows the medical team to access patients’ information including the medical care they received within seven days, and veritably shows whether the team members have read communication messages. After pilot adoption, an online survey was conducted. (3) Results: A one-stop IPC platform was implemented and promoted for patient care. The online survey revealed that medical team members have high positive appraisal of the platform. It also pointed out that resistance to change among the medical team still has a significant impact on behavioral intention. (4) Conclusions: The interprofessional collaboration platform was recognized by the medical teams of Chi Mei Medical Center as an effective and convenient tool for assisting clinical decision making. However, actions to reduce user resistance to change and encourage collaboration among team members still need to be continued. Shared decision making within physicians and patients will be valuable to develop in the platform in the future.

## 1. Introduction

Providing healthcare requires a set of highly specialized skills; teamwork among the medical team is also necessary to provide holistic care. Medical errors at any level may cause mental and physical harm to patients. To minimize mistakes, medical care requires a high level of interprofessional collaboration (IPC). Medical team members must practice interdependence and mutual collaboration to ensure the provision of optimal patient care. The World Health Organization (WHO) defines IPC as ‘multiple health workers from different professional backgrounds working together with patients, families, caregivers, and communities to deliver the highest quality of care’ [1]. Insufficient team work and communication is one of the main causes for medical errors and/or near misses [2,3]. Power imbalance, insufficient knowledge of different posts’ responsibilities, and the problems brought by friction in a professional field may influence the success of IPC [4,5]. Consequently, the Joint Commission on Accreditation of Healthcare Organization (JCAHO) has always emphasized the need to “improve staff communication” as one of the National Patient Safety Goals to reduce the risk of patient mismanagement; it is expected that improving the effectiveness of communication with caregivers and among the medical team will reduce the security risk of the patients and will ensure medical care quality [6,7].

A medical team, often composed of a large number of health professionals with different fields of expertise, includes but is not limited to physicians, pharmacists, nurses, lab technicians, occupational, physical, and speech therapists, and social workers. In addition, a health professional may be specialized in a specific discipline, such as surgery, internal medicine, orthopedics, anesthesiology, medical imaging, pathology, etc. Oftentimes, team members have different perspectives on the same medical situation depending on their field of specialization. For instance, a surgeon and an internist may have very different opinions on a certain diagnosis, which will render different treatment options; thus, collaboration is needed from different healthcare professionals in order to devise an optimal treatment plan. It is understandable that team members with different expertise and standpoints may have divided opinions on the same medical situation. A medical team often deals with emergencies, which urge them to respond quickly and reflexively hindering sufficient team communication in decision-making; regrettably, this may lead to medical mistakes. According to the Swiss Cheese Model, every health worker may make mistakes, but mistakes can be prevented if team members are attentive [8]. Consequently, it is crucial that each member of the medical team upholds mutual respect, values communication, and appreciates other’s viewpoints and standpoints.

IPC emphasizes that cross-disciplinary professional collaboration produces insights that are superior to individual professional fields. Its application in medical education and learning called interprofessional education (IPE) has been widely valued and practiced. According to WHO, ‘IPE occurs when students from two or more professions learn from and with each other to enable effective collaboration and improve health outcomes.’ IPC in education can specifically realize learning in schools and clinical settings (e.g., [9]); the assistance of technology (e.g., e-learning or virtual conference) has also been widely recognized by learners (e.g., [10]). In the development of IPE in the clinical setting, it is important to consider cross-disciplinary learning because the patient’s condition is often multi-faceted and requires cooperation among different professionals. Brault and colleagues [11] conducted an IPE pilot study in four healthcare settings and discussed how interprofessional learning activities (ILAs) were implemented during students’ professional practicum and how informatics was adopted in the implementation. The study validated the relevance of ILAs and the value of promoting professional exchanges between students of different professions, both in academia and in the clinical setting. The study also revealed that informatics appears to offer opportunities for connecting students from different professions and contributes to their professional development. Gurevich and colleagues proposed a combined synchronous and asynchronous collaborative e-platform curriculum which was found to be beneficial for the integrated training of hospital language therapy and nursing care; also, it can be flexibly extended to the care of other diseases [12]. IPC has been observed widely in the field of medicine; medical research conducted by health professionals with various field of specialization have been found to have good results, especially for high-risk or complex diseases, such as oncology [13]. 

Reeves [14] suggested that IPC has three main interventions namely: (1) education-based, (2) practice-based, and (3) organization-based. Among them, practice-based IPC interventions may be beneficial when incorporated through a tool or routine practice to improve the effects of IPC. As medical equipment becomes more advanced and complex and people becoming more health-conscious, convenient and instant modes of communication should be urgently provided to medical teams to ensure ease with teaching, learning, and decision-making. With communication technology advancement and mobile device popularity, it is practical that a computerized tool conducive for both medical team and patient communications is developed to support medical interventions. 

In providing patient care, an electronic platform is highly valuable to effectively promote cooperation and communication among cross-disciplinary teams. Only a few technology-based IPC for specific care delivery have been realized. For example, Heath and colleagues [15] developed the “Listening to you” communication tool for healthcare staff delivering pediatric care, allowing them to listen, respond, and incorporate parental concerns with their child’s medical plan in the hospital. In addition, Karlsudd [16] developed a cooperation system that facilitates communication and collaboration among caregivers and parents of disabled children, and proved its usefulness and ease-of-use. A patient-centered IPC platform for all kinds of health workers within a hospital, however, is quite rare (e.g., [17]). 

Therefore, the purpose of this study is to develop and evaluate a hospital-wide patient-centered IPC platform, which can significantly contribute to the knowledge of IPC for academic and practical application purposes.

## 2. Materials and Methods 

### 2.1. Project Development

Establishing an interprofessional collaboration and communication platform (hereinafter referred to as “IPC platform” or “platform”) will provide a patient-centered communication channel for healthcare workers. The platform will integrate the medical records and treatment recommendations of various health professionals from different disciplines for the care of the same patient. Presently, patient information is stored in separate hospital information systems (HIS) available per department. Through this platform, all records will be displayed in one page. It will help the user know who among the team has seen the patient notes, and to which medical department the member belongs. Further, this centralized channel will help each member become familiar with the other members of the medical team and will put all team members on a common ground. To develop the platform, Chi Mei Medical Center formed a group in May, 2017 who became in charge of the project. Led by the Superintendent’s Office, the group consisted of the following: members of the Medical Records Management Committee, Department of Surgery, Department of Internal Medicine, Emergency Department, Department of Anesthesiology, Chinese Medicine Division, Department of Nursing, Department of Rehabilitation, Department of Nutrition, Department of Social Services, Quality Management Center, and Department of Information Systems. The aim of the group was to give the medical team an information platform that will facilitate team communication, provide immediate medical treatment, and integrate the platform with the current systems being used in the hospital (i.e., HIS) to improve the efficiency of the medical team. It is hoped that the creation of a new interactive platform will give equal importance to each health discipline and strengthen the collaboration among the medical team.

### 2.2. Development of the IPC Platform

The development of the platform was headed by the Department of Information Systems of Chi Mei Center, and the method used was a software development life cycle (SDLC) [18], which includes core phases of system analysis, system design and system implementation. 

#### 2.2.1. IPC Requirement for Patient Care

**Figure 1:**
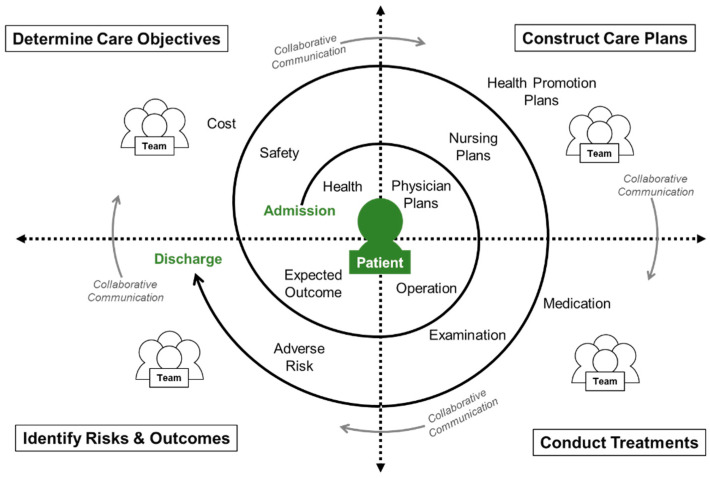
Interprofessional collaboration (IPC) spiral-shaped information flow for patient care.

Patient care is a cross-disciplinary team activity that can be simplified into a continuous cycle of four phases: (1) determining care objectives, (2) constructing care plans, (3) conducting treatments, and (4) identifying risks and outcomes. It continues in a spiral flow starting from the patient’s admission and ending at the patient’s discharge. During this period, medical experts must continuously cooperate and communicate the patient’s condition amongst each other to provide the patient with the best quality of medical care. Figure 1 outlines the IPC spiral-shaped information flow and presents the role and activities of each healthcare member in the four-phase patient care delivery system for a specific patient admitted in the hospital. The purpose of this study is to develop a comprehensive, computer-mediated platform for team collaboration to effectively support the existing HIS. It begins with the patient being the center of care; the platform integrates the existing electronic medical records, quickly grasping the patient’s current status, transmitting information in real time, and avoiding repeated input.

#### 2.2.2. System Analysis

The platform is intended as a communication channel for the medical team to allow them to communicate quickly and conveniently, with the goal of improving medical care quality. Before establishing the platform, the developers interviewed all the departmental heads to determine the types of information each department needs to convey with other team members, how the needed information should be displayed on the platform, how the receiver of the message could easily operate the platform, and how the information could be used in teaching and learning among team members.

- Review of all existing HIS

It is a burden for healthcare workers to input the same information repeatedly, especially when they already have a heavy workload. Since information about the same patient is scattered over various HIS, it is necessary to identify the kind of information a medical team needs to communicate and whether the information provided in the existing systems are complete. After all the HIS were reviewed, it was found that most necessary patient information can be obtained from the HIS of main hospital records, and emergency, nursing, pharmacy, nutrition, respiratory, rehabilitation, and anesthesiology departments. Other information can also be obtained from the medical examination record and records of other members of the medical team, such as psychotherapists and social workers. This is summarized in Table 1. Further appraisal showed that there are over 40 HIS in the hospital. Having several separate HIS makes it difficult for the medical team to communicate amongst each other; thus, this necessitates modest revision and the development of the platform so that all HIS would allow the exchange of information.

- Optimization of Information Presentation

After the existing HIS were reviewed, the developer was able to recognize the type of information the health workers expect to see on the platform and the layout of the page that would facilitate ease of reading. The existing systems are often limited to each department, and their pages have different layouts, colors, labels, and methods of information presentation. Each department was consequently provided with an adjusted page, which was then used in the platform. 

**Figure 2:**
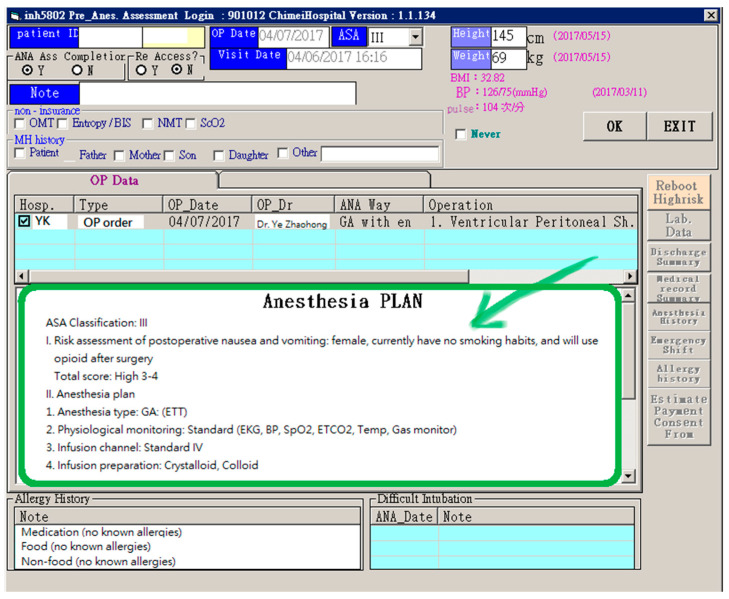
The adjustment of the layout of the anesthesia plan (optimized in the existing anesthetic system).

Figure 2 shows the HIS of the anesthesiology department as an example. On the interface of the anesthetic plan, the physicians are required to mark applicable items for each patient. This interface makes it difficult for other members of the medical team to understand the information at first glance, especially those belonging in a different department. Therefore, the developer adjusted and optimized the layout of the old interface for ease of reading (shown at the bottom of Figure 2). 

#### 2.2.3. System Design

- System Function Design

**Figure 3:**
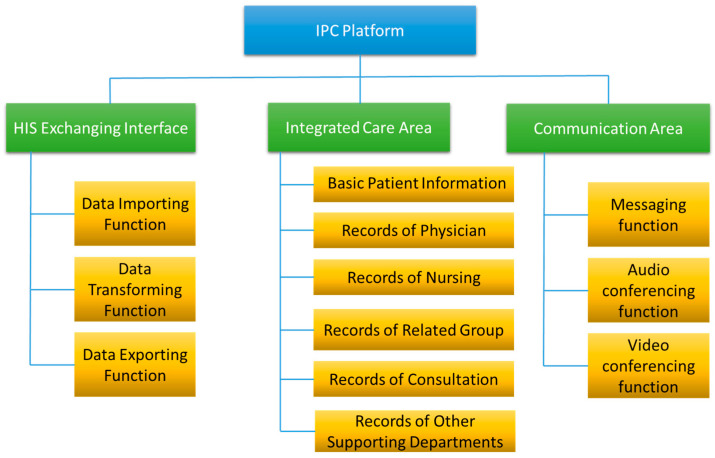
Functional architecture of the IPC platform.

The system functions were designed based on two major principles: first, clinical information scattered over different systems shall be integrated into one page, including the physician ordering system, nursing system, examination and laboratory systems, etc.; and second, the related departments shall check the output of their HIS and decide which information will be displayed on the platform. There are three major functional parts on the platform: (1) the HIS exchanging interface, (2) the integrated care area, and (3) the communication area. The detail functional architecture of the platform are shown in Figure 3.

(1) HIS Exchanging Interface

The existing HIS may have a variety of databases and data formats, so a data exchange mechanism between the platform and HIS is required. With functions of data importing, data transforming and data exporting, this platform can ensure the timeliness and consistency of information exchange and presentation with the existing HIS. Because medical records are regarded as legal documents, they are required to be worded carefully and confirmed with an electronic signature. Thus, we position this platform as a convenient communication channel for team communication, on which dialogue messages can be recorded, but not stored (exported) in the medical history master file directly. However, medical personnel can use the existing HIS (for example, the admission note system) to import the suggestion messages from the platform as references while documenting medical records. As a result, it makes medical personnel more willing to use it.

(2) Integrated Care Area

The integrated care area is presented in a spiral-shaped pattern with basic patient information at the center, and the medical departments participating in patient care are color-coordinated and stacked around the patient icon. Basic patient information includes a patient’s name, gender, age, blood type, admission date; records of the physician include the orders of the patient’s admission note, progress note, and order sheet; records of nursing include TPR/pains, nursing care records, nursing plans, and discharge preparing service; records of related groups include operation, anesthesia, examination and laboratory; records of consultation include the nutritional care group, hematology and oncology, infection control and rehabilitation; records of other supporting departments include physical therapy, occupational therapy, pharmacist care, nutrition screening and care, and respiratory therapy.

(3) Communication Area

**Figure 4:**
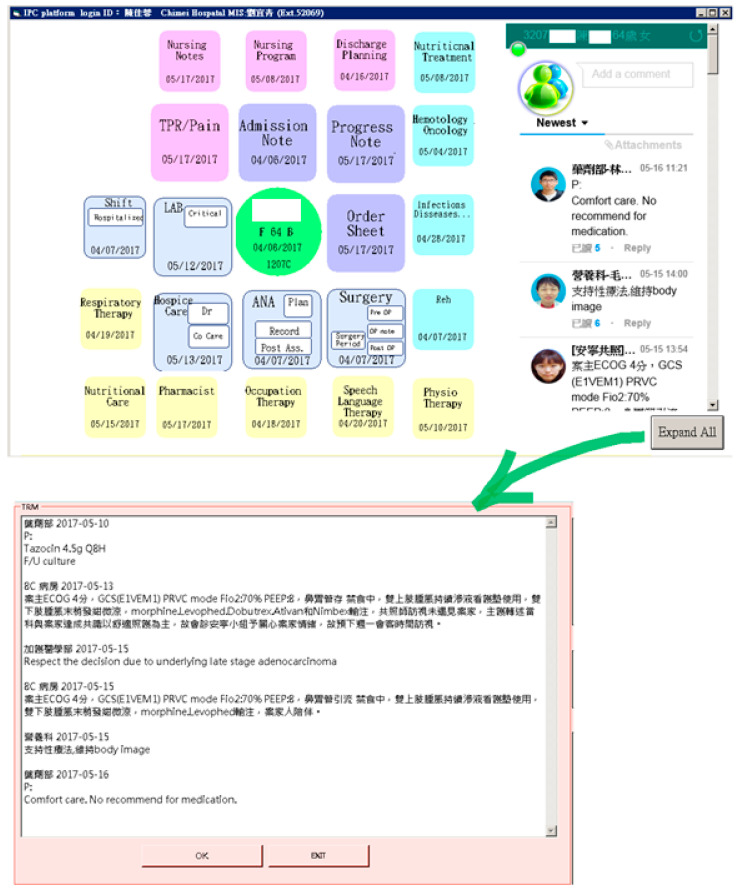
Master page with two functional areas, and the expanded page displaying the message history.

The records made on the existing HIS were synchronized with the communication area of the platform. Similar to the social application Line^®^, this area displays new messages sent within seven days, indicating when the messages were sent, what messages were sent, and whether the messages have been read. Moreover, the complete message history regarding the patient collected during the whole hospitalization can be displayed in a separate page by clicking the “expand all” button; team members who read the message and the department they belong to can be also verified (see Figure 4). In addition to text messaging, the platform also provides functions for audio and video conferencing.

- User Interface Design

**Figure 5:**
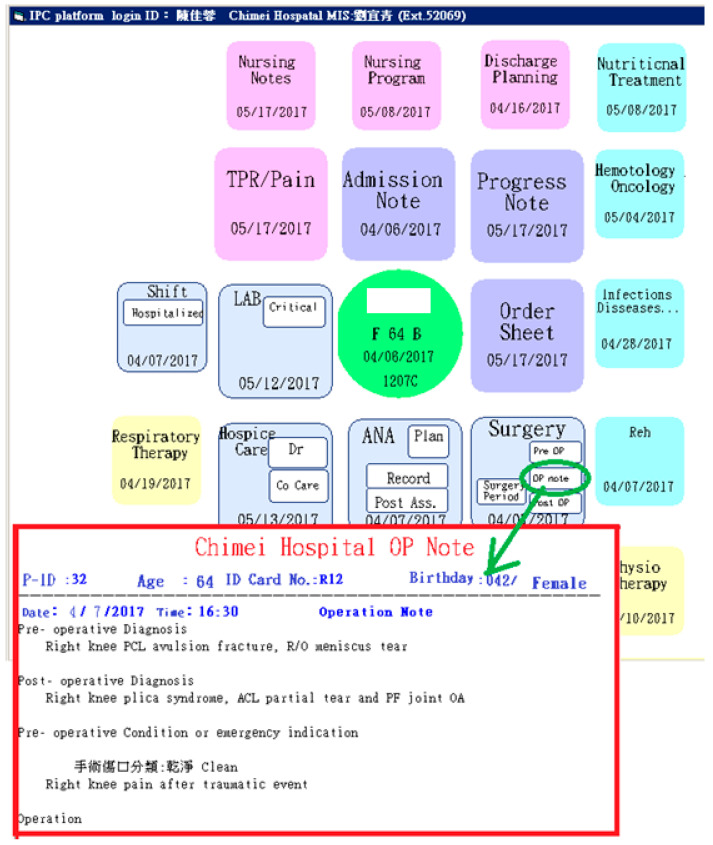
The spiral-shaped master page and a sample of a detailed page.

During the development of the platform’s layout, the developer considered whether all needed information should be displayed in only one page. After discussion among the project team members, it was decided that the departmental icons would be arranged in a spiral-shaped order and would have different colors to distinguish the different departments. The departments and the colors assigned to them are listed in Table 2, and the spiral-shaped interface is shown in Figure 4. The order in which the information is displayed on the platform was based on the hospitalization process of an in-patient. A separate page could be opened for specific information, such as surgical operation notes, and pre-operative, post-operative, or perioperative nursing records (See Figure 5). Icons are color-coordinated to the relative medical professions and displayed in a spiral-shaped order starting with the basic patient information at the center, which then radiates outward in a clockwise manner beginning with the core physician tasks (admission note, progress note, order sheet, etc.), followed by the care group tasks (operation, anesthesia, examination and laboratory, etc.), nursing tasks (nursing record, nursing plan, discharge preparation, etc.), care consultation tasks, and care supporting tasks (nutrition, pharmacy, physical therapy, respiratory therapy, etc.).

- System Architecture Design

The client-side of the platform is a Window-based application; this is listed in the menu of HIS portal and can be used by authorized health workers. Health workers can also fork the application in their HIS (through a button click). Meanwhile, the server-side of the platform is a separate database system installed in a PC server. Once health workers update the patient data (e.g., a physician prescribes a medication for his/her patient) through HIS, the data will be synchronized to the platform database, ensuring that all users can see the recent data input. The platform database (MariaDB^®^) was separated from the HIS database (Informix^®^) to reduce the impact on core front-end tasks (HIS tasks). Further, the virtual desktop system (Citrix^®^ solution) was adopted to allow the users to access the platform with their handheld devices or smartphones just like using a mobile application (APP).

#### 2.2.4. System Implementation

The platform is based on the simple concept of team members being able to have a general picture of the patient based on the information viewed in a single page. The platform will also indicate the relevant medical professionals who came in contact with the patient, and will serve as a tool for each team members to communicate directly. This is the so-called “one-stop” platform for all care members.

The platform integrates the medical records and treatment recommendations from different health workers who come from different departments for the same patient. MS Visual Studio^®^, a window-based development tool, was used to implement the pilot system. It was built for desktop and mobile operation modes. The desktop version was installed on PCs with full functions (for larger screen size), while the mobile version was installed on mobile devices (e.g., iPhone, iPad or other tablet PCs) with limited functions (for a smaller screen size).

## 3. System Evaluation

### 3.1. Clinical Significance

Chi Mei Medical Center started system testing in December, 2017. Every two weeks, a discussion on the use of the platform’s system was held and the system was then revised for further improvement. The platform was officially launched in May, 2018 and put into use thereafter. The framework of the computerized system is dynamic. All admitted patients had a spiral-shaped master page; the functional icons displayed on the page are the departments that take part in the care of the patient. In this way, the number of departments that participate in the patient’s medical care is known just by looking at the master page. Consequently, its instant communication functions allow the whole medical team included in the care to be easily alerted and informed. Although the healthcare workers are accustomed to using the previous separate HIS, they gradually became familiar with the platform. The log statistics (i.e., messages sent and read) on June, 2018 showed that there was an increase in the use of the platform, which indicates that members of the medical team are gradually accepting its use. (#Sent/#Read): consultation (6464/2881), palliative care (283/177), social workers (415/207), pharmacist (726/616), and nutritionist (1429/838). 

### 3.2. Education Training

**Figure 6:**
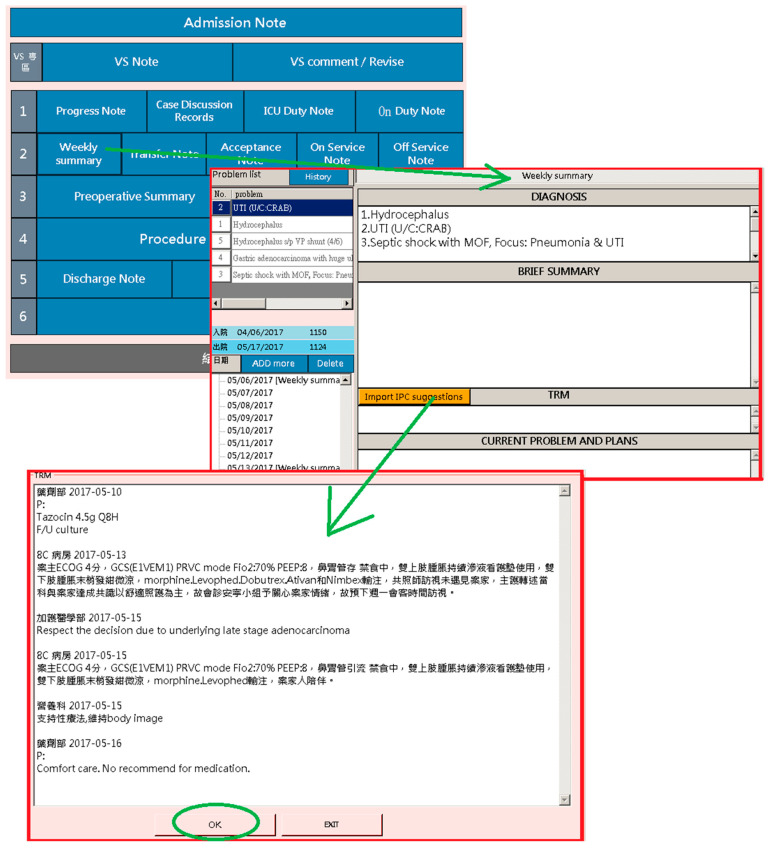
Weekly summary of a patient’s medical care records.

**Figure 7:**
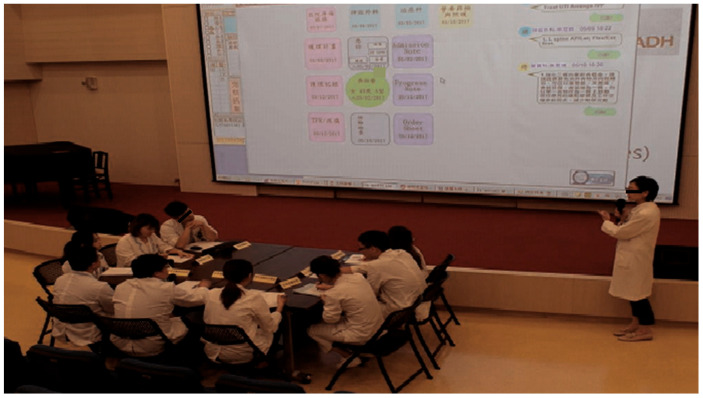
The platform being used during case discussion.

Physicians are required to document medical records of the admitted patient. Patient documentation must include the advice and opinions suggested by other team members responsible for patient care, which are integrated in the “Weekly Summary of Admission Note System” of the platform. In this way, a general picture of the medical care that the patient received in the past week is presented. In the “Team Resource Management (TRM)” section of the weekly summary record, there is a “Retrieve Latest Advice from Other Departments” button, from which healthcare workers can obtain the latest information about the patient from the platform. As shown in Figure 6, the TRM records the latest basic information of the patient. The users are allowed to edit the information in the TRM section; this provides junior trainees, who are unfamiliar with team collaboration, an easy way to learn about joint effort and cooperation among different medical departments, giving them an idea of a holistic patient-centered approach to medical care. In addition, the platform could be used during routine case teaching and discussion, in which healthcare workers from different departments participate. Through this, data can be easily retrieved (see Figure 7), as opposed to going through all the separate HIS of each department; thus, teaching and learning are facilitated by the platform.

### 3.3. Team Members’ Acceptance and Resistance

#### 3.3.1. The Evaluation Model and Survey Design

With respect to information management, most of the previous studies on the acceptance or adoption of innovation technologies were carried out from “positive” perspectives, such as perceived usefulness and perceived ease of use through the famous technology acceptance model (TAM) [19]. As the application of information technologies is always influenced by both positive and negative factors, it is necessary to validate both to gain a complete understanding [20]. Thus, the present study utilized a simplified model comprising of two positive constructs, perceived ease of use and perceived usefulness, and a negative construct of resistance to change to evaluate healthcare team members’ intention towards the use of the IPC platform. Perceived usefulness (PU) refers to the extent to which a healthcare team member believes that using the IPC platform would enhance their care performance; perceived ease of use (PEOU) refers to the extent to which a healthcare team member believes that using the IPC platform would be free of effort; and behavioral intention (BI) refers to the strength of a healthcare team member’s intention to use the IPC platform [21]. The PU was measured using four items (e.g., “Using IPC platform will improve my work quality”), the PEOU using three items (e.g., “The IPC platform is easy to use allowing me to finish my work”), and BI using four items (e.g., “I will frequently use IPC platform to assist my healthcare work”). Resistance to change (RC) refers to the extent to which a healthcare team member prefers to maintain the status quo despite the pressure to use the new IPC platform; it was measured using four items (e.g., “I do not want the IPC platform to change the way I interact with the other members of the medical team”) [22].

#### 3.3.2. Survey Procedure and Results

An online questionnaire survey on the use of the IPC platform was performed to get feedback from pilot users. Convenience sampling was used to obtain survey participants. A total of 108 valid questionnaires were retrieved; participants included 35 respiratory therapists, 22 nurses, 21 nursing assistants, 10 physicians, 8 rehabilitation therapists, 6 pharmacists, 3 nutritionists, 2 quality managers, and 1 worker from another department. The results presented in Table 3 show that the IPC platform has high PU (mean = 4.4), PEOU (mean = 4.1), and BI (mean = 4.3); and low RC (mean = 2.8). These indicate that the respondents have high appreciation of the platform. 

Before causal relationship analysis was conducted, the data obtained from the respondents were assessed using three tests: reliability, convergent validity, and discriminant validity (see Table 3). Cronbach’s α for each of the constructs was greater than 0.9, exceeding the suggested cut-off value of 0.7, and the composite reliability (CR) of all constructs exceeded the suggested cut-off value of 0.6. These results indicate that the measurements satisfied the reliability criteria [23]. The average variance extracted (AVE) value for each construct was beyond 0.7, exceeding the cut-off value of 0.5, which suggests satisfactory convergent validity [24]. Additionally, as shown in Table 4, none of the construct intercorrelations exceeded the square root of the AVE for each construct, establishing discriminant validity [24]. Overall, all of the constructs in this study exhibited sufficient convergent and discriminant validity, indicating that this model has substantial predictive power.

**Figure 8:**
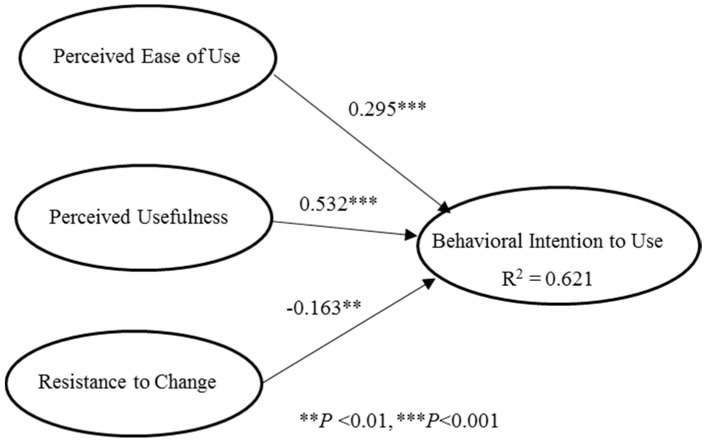
Partial least squares (PLS) path analysis results. (Note: values on the lines denote path coefficients of the causal relationships).

After confirming the reliability and validity of the model [24], the partial least squares (PLS) technique was used to evaluate causal relationships [25]. Figure 8 presents the path coefficients of the causal paths showing that all factors significantly influenced healthcare team members’ intention to use the IPC platform and jointly explained 62.1% of the variance. 

## 4. Discussion 

Clear, direct, and interactive communication is an important topic for healthcare reform. Chi Mei Medical Center has established the needed HIS for each of its departments; to improve its overall information system, the IPC platform was developed while integrating all the separate HIS. Aside from connecting all the HIS of each department, the platform allowed the members of the medical team to interact with each other through instant messaging using its mobile application. It is hoped that by providing the medical team with a tool that allows easy communication, team collaboration and practical training will be improved. 

Since the platform presents patient-related information on one screen page, it saves the user a lot of time. Instead of switching between different departmental information systems, all the needed information can be easily retrieved and viewed. Having a comprehensive understanding of the patient status by being equipped with needed information enhances clinical decision-making, which leads to better quality of medical care. Another advantage of the platform is that it provides a convenient communication channel for the members of the medical team to discuss patient data, saving them a lot of time. For example, a formal group consultation usually requires a day or more to be completed, and now using the platform, team members may gain appropriate responses from each other within half an hour (with the platform’s mobile application).

### 4.1. Comparison with Related Research

A variety of electronic tools for specific diseases or populations have been realized into practice, but a patient-centered IPC platform meeting a variety of health workers’ requirements within a hospital is still rare. This study compared Chi Mei Medical Center’s IPC platform with the IPC computer-mediated platform called “Care Connector” which was developed in a community teaching hospital in Canada [17]. This is summarized in Table 5.

Trillium Health Partners (THP) is a large community teaching hospital in Canada. They designed and implemented Care Connector using Agile software development methodology at a high user involvement level as a healthcare delivery tool for cross-disciplinary medical personnel in their hospital, providing quick and precise patient data. A total of 36 software programs were released during the first two years of its actual clinical use. As shown in Table N, our platform is superior to Care Connector because it presents the complete treatment process of a patient. Further, our platform fully integrates all existing HIS and allows members of the medical teams to input, view, and edit all current information of a patient they are treating together, providing real-time, interactive communication. This shows that our platform is more conducive in ensuring the completeness and sequence of care delivery. In addition, the platform supports synchronous communication (video telephony) and mobility (mobile application version), which are excellent innovations. Both platforms are worthy of reference for future development of medical IPC platforms.

### 4.2. Innovative Features of the IPC Platform

The one-stop IPC Platform is an innovation in clinical practice with the following features:

- Patient-centered platform: this platform is oriented towards the needs of the patient and integrates the latest information and treatment suggestions from different healthcare personnel.
- A tool for improved decision-making: the platform provides physicians with integrated medical care records as reference, allowing them to make informed and efficient medical plans and decisions.
- Dynamic presentation of patient’s medical record: every patient is treated by a team of medical professionals. All information they provide in terms of the care being given to the patient can be viewed in the platform. Through this, the members of the medical team participating in the care of the patient during hospitalization are aware of each other’s presence and suggestions.
- Information transmission and reception: the platform allows the members of the medical team to know who among them have read the messages sent by either them or fellow members.
- Avoidance of repeated input: the information provided by any of the medical team in the HIS is synchronized in the platform, so repeated inputs can be avoided.
- An effective teaching aid: the team can use the platform during case discussions, formal teaching, or rounds. Instead of looking for related information scattered over several HIS, all information can be obtained from the platform, saving the users a lot of time.

### 4.3. Resistance to Change Cannot Be Ignored

The survey results demonstrated that users’ negative perception played a critical role on the adoption of the platform. Hospital managers should always think of ways on how to diminish healthcare workers’ resistance to innovation. It is suggested that the managers of Chi Mei Medical Center to continue to strengthening the function (usefulness) and convenience (ease of use) of the IPC platform and to let users feel that using IPC will allow them to easily grasp the complete status of each patient, helping them to improve the medical quality they provide and their peer communication. Further, they should encourage its use rather than make it mandatory for all. Finally, when more medical personnel are willing to communicate their patient care through the platform, the number of those who will be willing to try and use the platform will increase, helping to reduce other members’ resistance.

## 5. Conclusions

Interprofessional education (IPE) and interprofessional practice (IPP) value medical collaboration training and play an increasingly important role in medical care. In 2001, the American Institute of Medicine (IOM) advocated that the training of medical professionals should equip them with the ability to work in an interdisciplinary team [26] so they can deal with increasingly complicated medical situations [27]. In 2010, WHO published the “Framework for Action on Interprofessional Education and Collaborative Practice”, which discussed how IPE and IPP can be used to create healthy, collaborative, practice-ready settings, helping to improve healthcare quality [1]. The IPC platform, developed by Chi Mei Medical Center, has already achieved this goal of IPP. It not only improves communication among team members, but also integrates materials needed for medical training and teaching. On the basis of the current platform, the hospital will develop more functions, similar to Ellman’s work [28], to improve its application to IPE and achieve the goal set by WHO.

Although the IPC platform yielded great results, some limitations were noted. For instance, since the participants of the IPC platform’s survey were health workers from a single hospital, the extrapolation validity of the study may be insufficient. Additionally, as the survey questionnaires were completed through self-reporting, the common method bias or common method variance may have been produced.

This study suggests that more diverse healthcare students should be exposed to interprofessional learning earlier in their education [29]; a smart tool like our IPC platform will surely be of great help. Further, dimensions for a more objective evaluation should be included in future studies, such as healthcare quality, cost-effectiveness, and information retrieval speed, which can be measured through actual healthcare operation; this can further persuade other healthcare professionals to adopt the platform. Consequently, other perceptual inhibitors of the IPC platform, such as technostress [30], need to be explored. Dissatisfaction was expressed by clinical workers over the suitability of smart technology in different communication contexts [31]; thus, future interventions using advanced technology should be taken into consideration while further developing the IPC platform. Immediate decision support in cross-disciplinary cooperation and communication will also be a very promising development direction, as well as introducing artificial intelligence and big data computing features into the IPC platform [32]. Furthermore, a few cases of inter-hospital cooperative care for patients have raised the benefits of cost and quality [33]; more in-depth issues are called for exploration. Lastly, shared decision-making between the healthcare workers and the patient, that is, how the opinions of the patient in terms of one’s health condition will be considered by the medical team, during relevant decision-making is an important topic for future development of the platform.

## Figures and Tables

**Table 1:** Information that needs to be shared among the medical team members and their sources.

Items	HIS System
Basic Information (Name, gender, age, blood type, hospital admission, patient safety message i.e., palliative notes, vessel, vital sign, height/weight/BMI)	Patient Master Information System
Emergency triage, ER notes, ER progress note, physicians’ advice for emergency treatment, records of emergency treatment and nursing	Emergency System
Admission note	Hospitalization Recording System
Progress note	Progress Note Recording System
Order sheet	Physician Order Entry System
Pre-op, op note, post-op, perioperative nursing records	Operating Room System
Anesthetic plan, anesthesia notes, records of postoperative visit	Anesthesia System
Critical value, risk value, examination report, etc.	Examination and Laboratory Systems
Palliative care records	Palliative Care System
Vital signs	TPR and Pains System
Nursing record	Nursing Recording System
Nursing care plan	Nursing Care Plan System
Discharge planning	Discharge Plan System
Group consultation records with physicians from different departments	Consultation System
Physical, occupational, and speech-language therapy	Rehabilitation System
Pharmacist record	Pharmacy System
Nutrition screening and assessment records	Nutrition System
Records of respiratory care	Respiratory Therapy System
Social worker record	Social Worker System
Psychotherapy record	Psychotherapy System

Note. TPR denotes temperature, pulse and respiration.

**Table 2:** The functional categories and their corresponding color.

Functional Category	Icon Color	Example (from Figure 5)
Basic Patient Information	Green	Name, Gender, Age, Blood Type, Admission Date
Records of Physician	Purple	Admission Note, Progress Note, Order Sheet
Records of Nursing	Pink	TPR/pains, Nursing Record, Nursing Plan, Discharge Preparing Service
Records of Related Group	Lilac	Operation, Anesthesia, Examination and Laboratory
Records of Consultation	Blue	Nutritional Care Group, Hematology and Oncology, Infection Control, Rehabilitation
Records of Other Supporting Departments	Yellow	Physical Therapy, Occupational Therapy, Pharmacist Care, Nutrition Screening and Care, Respiratory Therapy

**Table 3:** Descriptive statistics of the criteria for the quality of the responses.

Construct	Mean	SD	CR	Cronbach’s Alpha	AVE
BI	4.29	0.71	0.96	0.95	0.86
PEOU	4.08	0.83	0.94	0.90	0.83
RC	2.75	1.05	0.96	0.90	0.87
PU	4.35	0.70	0.93	0.95	0.76

Note. SD denotes standard deviation; CR denotes composite reliability; AVE denotes average variance extracted; BI denotes behavioral intention; PEOU denotes perceived ease of use; RC denotes resistance to change; PU denotes perceived usefulness.

**Table 4:** Correlation matrix.

	BI	PEOU	RC	PU
BI	**0.93**			
PEOU	0.65	**0.91**		
RC	0.22	−0.02	**0.93**	
PU	0.74	0.65	0.10	**0.87**

Note. The bold numbers on the leading diagonal show the square root of the variance shared by the constructs and their measures.

**Table 5:** A Comparison with Care Connector [17].

Novelity	Care Connector	IPC Platform	Note
Patient-centered	Yes	Yes	For better care quality and reduced waste
Scale	Hospital-wide	Hospital-wide	Any kind of health worker can use it
Integrated existing HIS	Integration	Full integration	Incoperates all related care data together for a specifc patient and avoids repeated input data
Communication mode	Asynchronous	Asynchronous and synchronous	Provides functions seen in LINE mobile application
Basic video conference	Unsupported	Supported	Provides functions seen in LINE mobile application
Completeness and sequence confimation	Indirective	Directive	Provides a full view of a patient status in a page
Supported education training	Indirective	Directive	Easily retrieves suggestions of others from the platform for routine education activity
Mobility	Unsupported	Supported	Has a simplied version that can be accessed using a mobile application
User acceptance	High	High	Survey statistics
